# Straightforward Analysis of Sulfated Glycosaminoglycans by MALDI-TOF Mass Spectrometry from Biological Samples

**DOI:** 10.3390/biology11040506

**Published:** 2022-03-25

**Authors:** Lynn Krüger, Karina Biskup, Vasileios Karampelas, Antje Ludwig, Antje-Susanne Kasper, Wolfram C. Poller, Véronique Blanchard

**Affiliations:** 1 Charité-Universitätsmedizin Berlin, Corporate Member of Freie Universität Berlin, Humboldt-Universität zu Berlin, and Berlin Institute of Health, Berlin, Germany; lynn.krueger@charite.de (L.K.); karina.biskup@charite.de (K.B.); karampelas.vasileios@gmail.com (V.K.); antje.ludwig@charite.de (A.L.); antje.kasper@charite.de (A.-S.K.); wolfram.poller@charite.de (W.C.P.); 2 Institute of Diagnostic Laboratory Medicine, Clinical Chemistry and Pathobiochemistry, Charité-Universitätsmedizin Berlin, 13353 Berlin, Germany; 3 Department of Biology, Chemistry and Pharmacy, Freie Universität Berlin, 14195 Berlin, Germany; 4 Department of Cardiology and Angiology, Charité-Universitätsmedizin Berlin, 10117 Berlin, Germany; 5 DZHK (German Centre for Cardiovascular Research), Partner Site, 10117 Berlin, Germany; 6 Department of Vascular Surgery, Charité-Universitätsmedizin Berlin, 12200 Berlin, Germany

**Keywords:** glycosaminoglycans, mass spectrometry, chondroitin sulfate

## Abstract

**Simple Summary:**

Glycosaminoglycans (GAGs) are considered to be the most difficult type of glycoconjugate to analyze as they are constituted of linear long polysaccharidic chains having molecular weights reaching up to several million daltons. Structural analysis of glycosaminoglycans from biological samples is a long and work-extensive procedure due to the many preparation steps involved. In addition, so far, only few research articles have been dedicated to the analysis of GAGs by means of matrix-assisted laser desorption ionization time-of-flight mass spectrometry (MALDI-TOF-MS) because their ionization can be problematic due to the presence of labile sulfate groups. In this work, we present an optimized strategy to analyze GAG disaccharides via MALDI-TOF mass spectrometry, using a fast workflow that does not require purification after enzymatic cleavage. For the first time, we show that it was possible to identify the sulfation position in disaccharides obtained from GAG via fragmentation experiments. This proof of concept is illustrated via the analysis of chondroitin/dermatan sulfate disaccharides of atherosclerotic lesions, in which we were able to identify their monosulfation patterns.

**Abstract:**

Glycosaminoglycans (GAGs) are considered to be the most difficult type of glycoconjugates to analyze as they are constituted of linear long polysaccharidic chains having molecular weights reaching up to several million daltons. Bottom-up analysis of glycosaminoglycans from biological samples is a long and work-extensive procedure due to the many preparation steps involved. In addition, so far, only few research articles have been dedicated to the analysis of GAGs by means of matrix-assisted laser desorption ionization time-of-flight mass spectrometry (MALDI-TOF-MS) because their intact ionization can be problematic due to the presence of labile sulfate groups. In this work, we had the aim of exploring the sulfation pattern of monosulfated chondroitin/dermatan sulfate (CS/DS) disaccharides in human tissue samples because they represent the most abundant form of sulfation in disaccharides. We present here an optimized strategy to analyze on-target derivatized CS/DS disaccharides via MALDI-TOF-MS using a fast workflow that does not require any purification after enzymatic cleavage. For the first time, we show that MALDI-TOF/TOF experiments allow for discrimination between monosulfated CS disaccharide isomers via specific fragments corresponding to glycosidic linkages and to cross-ring cleavages. This proof of concept is illustrated via the analysis of CS/DS disaccharides of atherosclerotic lesions of different histological origins, in which we were able to identify their monosulfation patterns.

## 1. Introduction

Glycosaminoglycans (GAGs), produced by all mammalian cells, are major constituents of the extracellular matrix. As major components of tissues, they are either present in their free form or covalently linked to proteins. Sulfated GAGs are long acidic polysaccharides that usually bind to basic domains of proteins in a specific way [1] and regulate many cellular events, e.g., hemostasis, cell growth, migration and cell development [2]. Their complex sulfation patterns mediate very specific regulation of or binding to proteins and thus represent a molecular code [3]. These sulfation patterns can be severely altered under pathological conditions, such as inflammation and cancer, thereby affecting their function [4,5,6,7]. To better understand the structure–function relationships of sulfated GAGs and their resulting role in diseases, effective tools for the analysis of these highly complex glycans are desirable. Since the biosynthesis of GAGs is a non-template-driven event, which directly depends on the supply of glycosyltransferases, epimerases, sulfotransferases and activated monosaccharides, their analysis lags behind that of template-driven “omics” approaches such as genomics or proteomics. The development of high-throughput methods that would allow the analysis of complex sulfated GAGs from small amounts of biological material, such as biopsies or histological sections, is particularly important but challenging.

GAGs are composed of linear chains that are built out of repeating disaccharide units [8,9]. Their chain lengths range from nine to over 20,000 disaccharide units [10,11]. GAGs are structurally divided into five different categories, namely, chondroitin sulfate (CS), dermatan sulfate (DS), heparin/heparan sulfate, keratan sulfate and hyaluronan. CS is made of β(1-3)-*N*-acetylgalactosamine β(1-4)-glucuronic acid (β(1-3)-GalNAcβ(1-4)-GlcA) with various degrees of sulfation. As GAGs are made of extremely long polymer chains, bottom-up strategies are used to characterize their disaccharide compositions. Typical workflows consist of lengthy protocols, including GAG enrichment and enzymatic depolymerization, followed by numerous intermediate purification steps [4,12,13]. The enzymatic cleavage of CS (or DS) with chondroitinase ABC into disaccharides results in the unsaturation of internal GlcA (or iduronic acid, respectively) between the fourth and the fifth carbon atoms. All the disaccharides produced and/or used in this study contain this unsaturation, notated as Δ^4,5^. As a result, the isomericity between GlcA and iduronic acid is lost; therefore, CS and DS are analyzed together and cannot be differentiated from each other.

GAG analysis is very frequently performed via high-performance liquid chromatography, liquid chromatography coupled to electrospray ionization or ion-mobility mass spectrometry [4,13,14,15,16]. In addition, some workflows have been developed to analyze GAG from tissue biopsies [17,18]. Nevertheless, sample preparation from biological samples is usually time-consuming as it requires many purification steps [4,15] and data analysis is cumbersome. On the other hand, matrix-assisted laser desorption/ionization time-of-flight mass spectrometry (MALDI-TOF-MS) is a popular mass-spectrometric technique for the analysis of carbohydrates because it produces mainly singly-charged molecular species, which renders the data analysis simpler. In addition, sample measurement only takes a couple of seconds, which is shorter than other chromatographic techniques, for which a single sample measurement lasts between 15 min and 1 h. Unfortunately, desulfation of GAG disaccharides is frequently observed in MALDI-TOF-MS when free GAG disaccharides are measured because sulfate groups are labile. This problem can be overcome by first using a permethylation step for the free functional groups, followed by desulfation then acetylation of the remaining sulfation positions [19,20]. Recently, Wei et al. first labeled the reducing end with the fluorescent linker 2-amino-*N*-(2-aminoethyl)benzamide before performing muti-scale chemical derivatization steps, including N-acetylation, carboxyl amidation, permethylation and desulfation with a silylating reagent [21]. These methods are very robust and allow the unambiguous assignment of GAGs but are extremely time-consuming.

Another interesting approach is to minimize the issue of desulfation via the use of labeling reagents and/or specific matrices. The labeling reagent 1-pyrenebutyric hydrazide (PBH), which reacts with the reducing end of GAGs and glycans, was successfully applied to improve the ionization of di- and tetrasaccharides isolated from keratan sulfate, a form of GAG that is devoid of carboxyl groups [22]. The ionic liquid matrices 1-methylimidazole/hydroxycinnaminic acid and guanidinium salt of alpha-cyano-4-hydroxycinnamic acid allowed the measurement of up to decasaccharidic GAGs with minimal loss of sulfates [8,9]. In addition, the label 3-hydrazinobenzoic acid (3-HBA), used in combination with different MALDI matrixes, was very recently employed for on-target derivatization of *N*-glycans but has not been tested on GAG disaccharides so far [23].

In this work we present a fast and straightforward protocol to analyze CS/DS disaccharides without further purification. To do so, labeling with 3-HBA and matrix deposition were performed in a single step and sample amounts as low as 1 pmol could be measured. For the first time, we were able to differentiate the different sulfation positions of GAG disaccharides using MALDI-TOF/TOF-MS.

## 2. Materials and Methods

### 2.1. Chemicals, Reagents and General Considerations

Water-free dimethyl sulfoxide (DMSO) was obtained from Applichem (Darmstadt, Germany), acetonitrile (ACN) from VWR Chemicals (Dresden, Germany), acetic acid from Carl Roth (Karlsruhe, Germany) and methanol (MeOH) from Thermo Fisher Scientific (Schwerte, Germany). Chondoitinase ABC and chondroitin sulfate were obtained from shark cartilage; the chondroitin sulfate disaccharides Δ^4,5^HexA(α1-3)GalNAc (CS-0S), Δ^4,5^HexA(α1-3)GalNAc4S (CS-4S), 2-amino benzoic acid (2-AA), PBH, 3-HBA, 2,5-dihydroxybenzoic acid (DHB) and super-DHB were from Sigma-Aldrich (Taufkirchen, Germany). The chondroitin sulfate disaccharides Δ^4,5^HexA2S(α1-3)GalNAc (CS-2S), Δ^4,5^HexA(α1-3)GalNAc6S (CS-6S) were from Dextra (UK). Milli-Q grade water was produced using a MilliQ Plus water purification system (Millipore, Darmstadt, Germany). Labeling experiments were performed under a fume hood.

### 2.2. Patient Sample

Carotid endarterectomy (CEA) was used to obtain a carotid plaque from patients diagnosed with carotid artery stenosis. The project was approved by the ethics committee of the Charité-Universtitätsmedizin Berlin (EA 1/188/15). The patients gave written informed consent to participate. Experiments were performed in accordance with the 1975 Declaration of Helsinki as amended in 2013. CEA specimens were dissected into 5 mm thick slices and fixed with 4% (*v*/*v*) formaldehyde for 24 h at 4 °C. Decalcification was performed in 0.27 M EDTA pH = 7.40–7.44 for seven days at room temperature, with daily renewal of the EDTA solution. Finally, the tissue segments were embedded in paraffin and stored at 4 °C. To visualize morphology, serial 6-μm sections were mounted on glass slides, deparaffinized, and stained with Movat pentachrome stain.

### 2.3. On-Target Derivatization with 2-AA 

**Figure 1:**
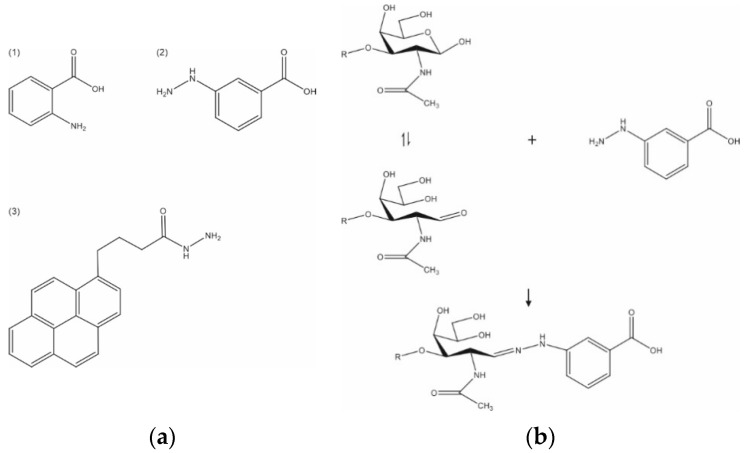
(**a**) Structure of the labels used in this study: (**1**) 2-AA, (**2**) 3-HBA, (**3**) PBH. (**b**) Derivatization scheme of the free reducing end of CS-0S using 3-HBA as an example. R: Δ^4,5^HexA.

The derivatization solution consisted of 2-AA (Figure 1(a1)) that was dissolved in methanol/acetic acid (9:1, *v*/*v*) at a concentration of 0.025 mmol/mL. Then, 1 μL of GAG disaccharide solution was applied onto a ground steel target (Bruker Daltonics, Bremen, Germany). After the spot dried, 1 μL of derivatization solution was applied onto the sample and the plate was heated for 2 h at 65 °C. After the plate cooled down, 1 μL of matrix solution (10 mg/mL super-DHB in 50% ACN) was pipetted onto the sample and the target was allowed to dry at room temperature.

### 2.4. On-Target Derivatization with PBH

Solution A comprised PBH (Figure 1(a3)) dissolved in methanol/acetic acid (10:1, *v*/*v*) at a concentration of 0.025 mmol/mL. The PBH derivatization solution consisted of 10 μL of solution A mixed with 90 μL of 80% MeOH (*v*/*v*). One μL of GAG disaccharide solution was first applied onto the target and left to air-dry. Subsequently, 1 μL of PBH derivatization solution was added on the dried spot and the plate was dried for 10 min at 70 °C. After the plate cooled down to room temperature, the previous step was repeated once more. Finally, the sample was covered with 1 μL of matrix solution and air dried.

### 2.5. On-Target Derivatization with 3-HBA

Solution A′ was made of 3-HBA (Figure 1(a2)) dissolved in methanol/acetic acid (9:1, *v*/*v*) at a concentration of 0.005 mmol/mL. The 3-HBA derivatization solution consisted of 0.2 M DHB dissolved in solution A′. The derivatization reaction was performed on the MALDI target. First, GAG disaccharide samples were applied. Afterwards, 1 μL of 3-HBA derivatization solution was deposited on the GAG spots and the target was left to dry for 30 min at 70 °C.

### 2.6. Preparation of GAG Disaccharides from Chondroitin Sulfate

Chondroitin sulfate from shark cartilage (100 µg) was dissolved in 50 µL 200 mM ammonium acetate (pH 7.7). Then, 10 µL chondroitinase ABC from *Proteus vulgaris* (10 mU/µL dissolved in 0.01% (*w*/*v*) BSA) and 40 µL MilliQ water were added to the sample, which was incubated overnight at 37 °C. After drying in a speedvac, salts were evaporated by adding 500 µL MeOH, then drying the sample in a vacuum concentrator. This step was repeated five times. The sample was redissolved in 500 µL MilliQ water and 1 µL of the solution was deposited on the MALDI target. Finally, the 3-HBA derivatization solution (1 μL) was applied on the dried sample and the target was left to dry for 30 min at 70 °C.

### 2.7. Preparation of GAG Disaccharides from Atherosclerotic Tissues

A paraffin-embedded human atherosclerotic tissue section of 50 µm was deparaffinized using the following washing steps: 2 × xylene, 2 × ethanol, 2 × water. The sample was then dried in a dessicator for 10 min. The enzymatic solution consisting of 25 µL 200 mM ammonium acetate (pH 7.7), 10 µL chondroitinase ABC (10 mU/µL) and 15 µL MilliQ water was applied to the sample and the sample was subsequently incubated overnight at 37 °C. Volatile salts were removed as described in Section 2.5. The sample was reconstituted in 200 µL MilliQ water. Then, 1 µL of the solution was deposited on the MALDI target and the 3-HBA derivatization solution (1 μL) was added to the dried sample and was left to dry for 30 min at 70 °C.

### 2.8. Mass Spectrometry

MALDI-TOF and -TOF/TOF mass spectra were recorded on an Ulfraflex III (Bruker Daltonics, Bremen, Germany) equipped with a Smartbeam laser and a LIFT-MS/MS facility. Spectra were recorded in reflector negative ionization mode in the *m*/*z* 400–800. LIFT-MS/MS fragmentations were performed using a collision energy of 29 kV. MALDI-TOF-MS spectra consisted of at least 2000 laser shots, whereas LIFT spectra were acquired with 1000 laser shots. CS-Structures were drawn with GlycoWorkbench software.

## 3. Results and Discussion

Aiming at analyzing GAG disaccharides using MALDI-TOF-MS, we first selected labeling reagents (Figure 1a) that were previously used by our group, as well as others, to derivatize other types of carbohydrates and tested their efficacy to derivatize CS disaccharides using CS-2S as a model substance. The labeling reaction is an irreversible reaction (Figure 1b) that enhances ionization via MALDI-TOF-MS for carbohydrates.

**Figure 2:**
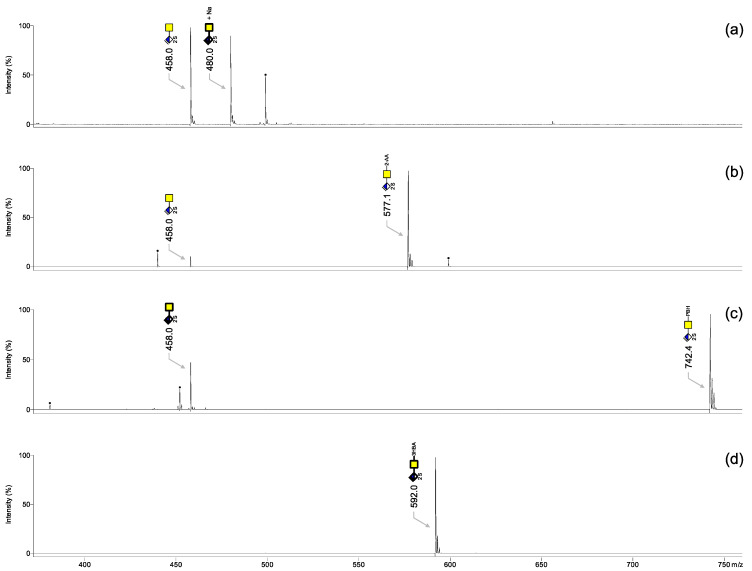
MALDI-TOF mass spectra of 100 ng CS-2S recorded in negative ionization mode: (**a**) non-derivatized CS-2S; CS-2S derivatized with (**b**) 2-AA, (**c**) PBH and (**d**) 3-HBA. All molecular ions are present in their [M − H]^−^ and/or [M + Na − 2H]^−^ form. 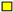, GalNAc; 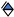, ΔHexA; S, sulfate. The digit preceding S indicates the sulfation position. Black dots correspond to non-GAG contaminants.

Figure 2a shows a MALDI-TOF mass spectrum of 100 ng non-labeled CS-2S in negative ionization mode. Both deprotonated (*m*/*z* 458.0) and sodiated forms (*m*/*z* 480.0) were observed with comparable intensities. A contamination peak at *m*/*z* 498.9 was present as well. Derivatization with 2-AA and PBH led to single deprotonated peaks but also to incomplete reactions (Figure 2b and Figure 2c, respectively). Afterwards, derivatizations with 3-HBA, performed as described in Section 2.5, led to single ionization peaks (Figure 2d). We also tested a concentration of 3-HBA as high as 0.025 mmol/mL, DHB concentrations varying from 0.5 M to 3 M, and a temperature of 60 °C, as well as two consecutive reactions with 3-HBA (data not shown). The intensity of MALDI-TOF mass spectra was the highest with a single on-target reaction using 0.005 mmol/mL 3-HBA and 2 M DHB at a reaction temperature of 70 °C, which are among the optimal conditions reported by Zhao et al. for the labeling of *N*-glycans [23].

**Figure 3:**
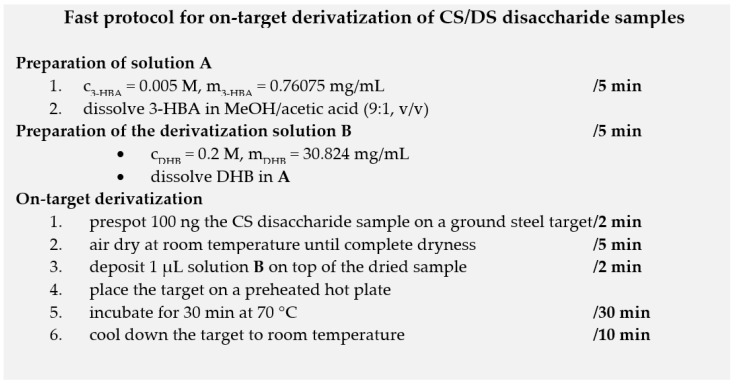
Analytical workflow used to prepare CS/DS disaccharide samples prior to MALDI-TOF mass spectrometric analysis.

The protocol of derivatization with 3-HBA is summarized in Figure 3 and takes only 1 h. A complete reaction could be achieved, probably because 3-HBA is a rather linear molecule compared to 2-AA and PBH (Figure 2d). On the contrary to the keratan sulfate disaccharides reported by Zhang and coworkers [22], labeling experiments with PBH did not give rise to the total conversion of free CS disaccharides. We also found that Girard T and Girard P reagents were not suitable to derivatize free CS disaccharides (data not shown). In other words, these workflows reported for *N*-glycans [24,25,26] were not transferrable to CS disaccharides.

**Figure 4:**
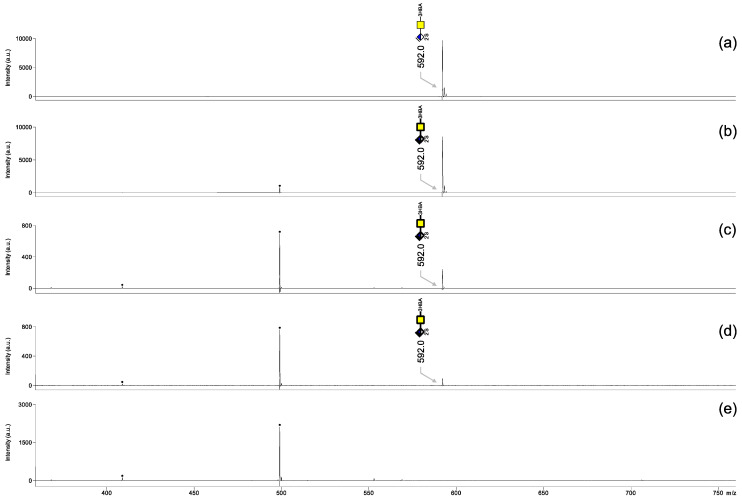
MALDI-TOF mass spectra of CS-2S derivatized with 3-HBA on target and measured in negative ionization mode. (**a**) 100 ng, (**b**) 10 ng, (**c**) 1 ng, (**d**) 0.5 ng and (**e**) 0.1 ng. All molecular ions are present in their [M − H]^−^ form. 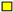, GalNAc; 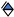, ΔHexA; S, sulfate. The digit preceding S indicates the sulfation position. Black dots correspond to non-carbohydrate contaminants.

Next, we tested the limit of detection of 3-HBA-labeled CS-2S. To this end, 100 ng, 10 ng, 1 ng, 0.5 ng and 0.1 ng CS-2S were applied as single spots on the MALDI target; then the 3-HBA solution was added and incubation was performed for 30 min at 70 °C. MALDI-TOF mass spectra were recorded in negative ionization mode (Figure 4) without any further cleanup. The 100 ng, 10 ng, 1 ng and 0.5 ng samples gave satisfactory signals, whereas the 0.1 ng sample gave a poor signal. Therefore, the limit of detection was as low as 0.5 ng for CS-2S, which corresponds to approximately 1 pmol of sample. This is ten times lower than the amounts reported for free heparin sulfate oligosaccharides when measured using an ionic liquid matrix [9].

**Figure 5:**
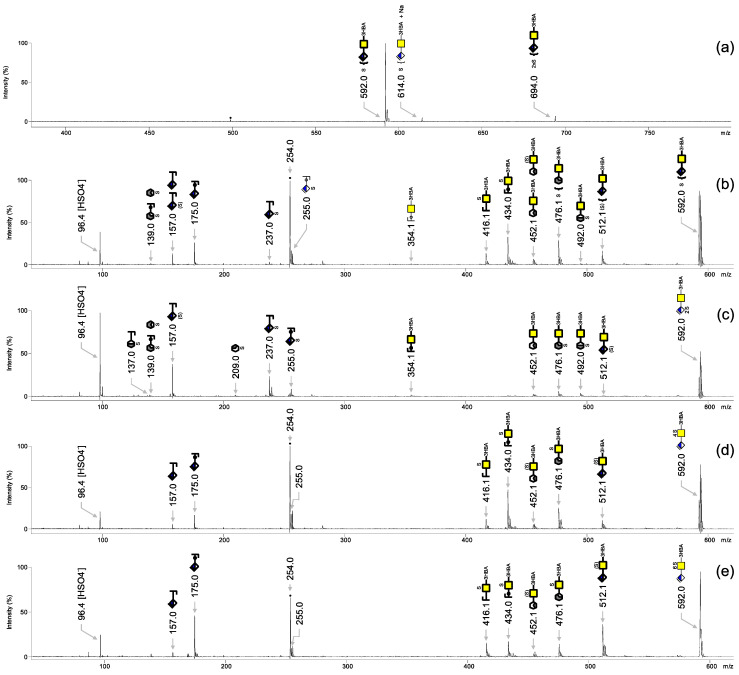
(**a**) MALDI-TOF mass spectrum of CS-disaccharides released from chondroitin sulfate from shark cartilage and derivatized on-target with 3-HBA. (**b**) MALDI-TOF/TOF mass spectrum of the monosulfated peak at *m*/*z* 592.0 from the MALDI-TOF mass spectrum obtained from shark cartilage shown in (**a**). MALDI-TOF/TOF mass spectra of the monosulfated peak at *m*/*z* 592.0 from (**c**) CS-2S, (**d**) CS-4S and (**e**) CS-6S. Mass spectra were recorded in negative ionization mode. All molecular ions are present in their [M − H]^−^ form. 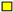, GalNAc; 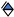, ΔHexA; S, sulfate; (S), absence of sulfate on the fragment. The digit preceding S indicates the sulfation position, • indicates contaminants. GlycoWorkbench software was used to represent and assign fragment ions.

A commercially available chondroitin sulfate polymer was digested into disaccharides using chondroitinase ABC, and volatile salts were removed with methanol as described in the Section 2, then derivatized on target with 3-HBA. MALDI-TOF mass spectra were recorded in negative ionization mode. The MALDI-TOF mass spectrum is presented in Figure 5a, in which monosulfated and disulfated CS-disaccharides were detected. The monosulfated CS-disaccharides ionized mostly in their [M − H]^−^ forms and traces of sodiated [M + Na − 2H]^−^ ions were found as well. The disulfated CS-disaccharides were measured only in the form of [M − H]^−^ ions.

Next, the monosulfated CS-disaccharide peak at *m*/*z* 592.0 was fragmented by means of MALDI-TOF/TOF-MS (Figure 5b) and compared with the MALDI-TOF/TOF-MS spectra of the commercially available disaccharide standards CS-2S, CS-4S and CS-6S (Figure 5c–e, respectively, and Table 1). Diagnostic fragments were assigned using the GlycoWorkbench program [27,28,29] and reported using the nomenclature of Domon and Costello [30]. The MALDI-TOF/TOF mass spectrum of CS-2S at *m*/*z* 592.0 was characterized by the signature fragments of glycosidic linkages at *m*/*z* 209.0 (^1,5^A_1_ fragment), 237.0 (B fragment), 354.0 (Y_1_ fragment) and the ^2,5^X_1_ cross-ring fragment at *m*/*z* 492.0 (Figure 5c). Regarding CS-4S and CS-6S, the diagnostic fragments had identical masses but their proportions were different: in CS-4S, the Y_1_ fragment at *m*/*z* 434.4 had a higher intensity than the ones at *m*/*z* 175.0 (C fragment) and 512.1 (loss of sulfate), whereas the opposite was the case in CS-6S. The fragment at *m*/*z* 255.0 in CS-4S and CS-6S was a contamination peak, rather than a fragment stemming from the rearrangement of Δ^4,5^HexA with a sulfate group, as this phenomenon was not reported in the past. As a result, through comparison of the MALDI-TOF/TOF mass spectra of CS obtained from disaccharide standards with that of CS obtained from shark cartilage, it can be concluded that the sample contained small amounts of CS-2S and high proportions of CS-4S and CS-6S disaccharides.

By comparing the fragments resulting from the MALDI-TOF/TOF-MS analysis of heparin sulfate oligosaccharides [9] and from the ESI-ion trap MS analysis of keratan sulfate oligosaccharides [31] that were measured in their free form, the use of the 3-HBA label at the reducing end in this work resulted in higher diagnostic fragments containing the reducing end, namely, X, Y and Z fragments, especially in the disaccharides CS-4S and CS-6S. Using LIFT fragmentation, we obtained cross-ring fragments as abundant as the ones obtained via electron detachment dissociation using the more advanced Fourier transform mass spectrometry [32,33]. In the MALDI-TOF/TOF mass spectrum of the parent ion *m*/*z* 592.0 of CS-6S, a high loss of sulfation was observed at *m*/*z* 512.1 compared to the MALDI-TOF/TOF spectra of CS-4S and CS-2S. This is most likely due to the fact that the C6 position is more labile than positions C4 and C2. In our fragmentation experiments, only cross-ring fragments were reported at the non-reducing end; cross-ring fragmentation of the reducing end did not occur. This was probably due to the labeling of the reducing end, which causes stabilization.

**Figure 6:**
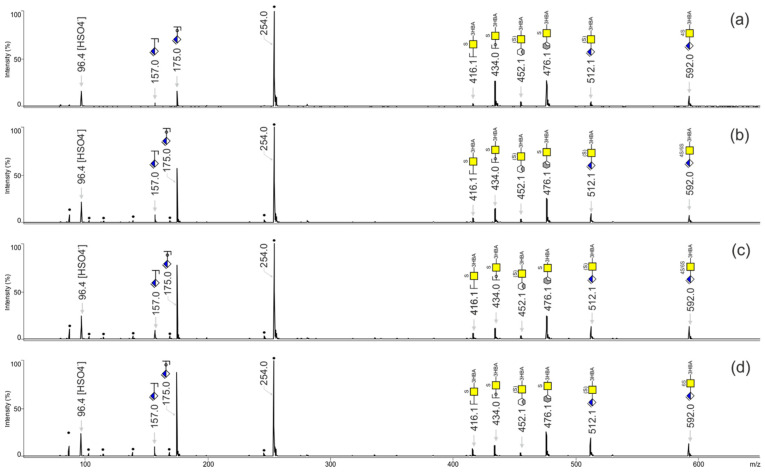
MALDI-TOF/TOF mass spectra of the monosulfated peak at *m*/*z* 592.0 from the MALDI-TOF spectrum obtained by mixing CS-4S and CS-6S in the following proportions: (**a**) CS-4S, (**b**) CS-4S/CS-6S (2:1), (**c**) CS-4S/CS-6S (1:2); (**d**) CS-6S. 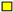, GalNAc; 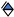, ΔHexA; S, sulfate; (S), absence of sulfate. The digit preceding S indicates the sulfation position; • indicates contaminants. GlycoWorkbench software was used to represent and assign fragment ions.

The MALDI-TOF/TOF mass spectrum of CS-2S was characterized by a unique diagnostic fragment at *m*/*z* 237.0. On the other hand, those of CS-4S and CS-6S were qualitatively identical but the intensities of the signals at *m*/*z* 434.0 and *m*/*z* 512.1 showed different proportions: CS-4S had a higher abundant signal intensity at *m*/*z* 434.0 and a low abundant signal intensity at *m*/*z* 512.1, whereas the opposite was the case for CS-6S. In order to confirm this, we mixed CS-4S and CS-6S standards in different proportions and recorded the MALDI-TOF/TOF mass spectra at *m*/*z* 592.0, as shown in Figure 6a–d. The ratio between the signals at *m*/*z* 434.0 and 512.1 varied with different isomer proportions.

**Figure 7:**
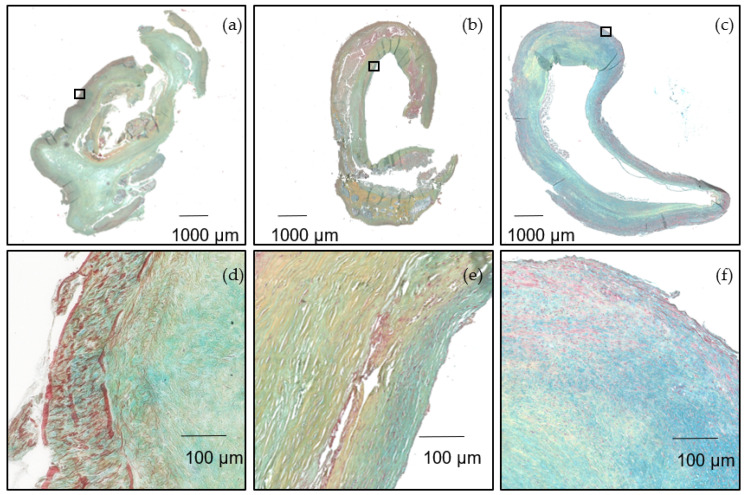
(**a**–**f**) Movat pentachrome stain of the histological sections of CEA specimens highlighting collagens (yellow) and GAG (blue). (**a**,**b**) late fibroatheroma, (**c**) fibrous plaque, (**d**–**f**) higher magnification showing alcian blue positive areas, indicating local accumulation of GAGs.

Finally, we analyzed three atherosclerotic lesions of different histological origin. The CEA specimens represented two late fibroatheroma and one fibrous plaque, as shown by histological analysis after Movat pentachrome staining (Figure 7e,f). Alcian-blue-positive areas showed the local distribution of GAGs (Figure 7e,f).

**Figure 8:**
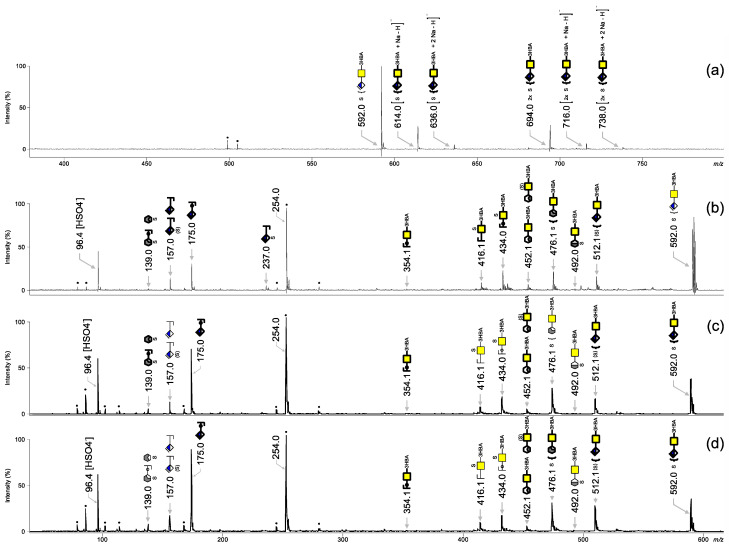
Representative MALDI-TOF mass spectrum of (**a**) CS-disaccharides released from chondroitin sulfate from human atherosclerotic lesions derivatized on-target with 3-HBA. MALDI-TOF/TOF mass spectra at *m*/*z* 592.0 of (**b**) the lesion shown in Figure 7a; (**c**) the lesion shown in Figure 7b; (**d**) the lesion shown in Figure 7c. 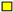, GalNAc; 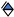, ΔHexA; S, sulfate; (S), absence of sulfate on the fragment. The digit preceding S indicates the sulfation position. GlycoWorkbench software was used to represent and assign fragment ions.

Atherosclerotic tissues were digested with chondroitinase ABC and volatile ammonium and acetate salts were removed with methanol. CS disaccharides samples were subsequently derivatized with 3-HBA on-target. A representative MALDI-TOF mass spectrum recorded in negative ionization mode is presented in Figure 8a. Monosulfated and disulfated CS/DS disaccharides ionized mostly in their [M − H]^−^ form (*m*/*z* 592.0) but [M + Na − 2H]^−^ and [M + 2Na − 3H]^−^ ions, at *m*/*z* 614.0 and 636.0, were observed as well. The disulfated CS/DS disaccharides ionized mostly in their [M + Na − 2H]^−^ form (*m*/*z* 694.0), but the additional sodium adducts [M + 2Na − 3H]^−^ and [M + 3Na − 4H]^−^ were detected at *m*/*z* 716.0 and 738.0, respectively. The monosulfated CS disaccharide peak at *m*/*z* 592.0 was further analyzed by means of MALDI-TOF/TOF in order to assess the CS disaccharide isomers present in human arteriosclerotic tissues.

Observing the ratios between the peaks at *m*/*z* 476.1 and *m*/*z* 512.1, we found that the two late fibroatheroma (Figure 8a,b) contained a higher proportion of CS-4S than CS-6S, whereas CS-6S was more abundant in the fibrous plaque (Figure 8c), with a CS-4S/CS-6S ratio of about 1:2. Although no general statement can be derived from this due to the minimal sample size, the presented method was capable of detecting varying proportions of monosulfated CS isomers, making it a valuable tool for future studies with large sample sizes.

## 4. Conclusions

We have presented, for the first time, the digestion and on-target derivatization of released CS/DS disaccharides without further purification steps. The workflow was used to analyze CS disaccharides from reference disaccharides as a proof of concept on atherosclerotic lesions. We also showed that MALDI-TOF/TOF fragmentation of monosulfated CS disaccharides allowed the assignment of the sulfation position. The approach presented here is particularly attractive in view of future (high-throughput) screening of biological samples, which mostly contain monosulfated GAGs.

## Figures and Tables

**Table 1:** Fragment ions detected in MALDI-TOF/TOF experiments for the monosulfated parent ion at *m*/*z* 592.0 in the CS disaccharide standards CS-2S, CS-4S and CS-6S. Fragment ions were named according to the nomenclature established by Domon and Costello [23]. (S) stands for the loss of a sulfate group during the fragmentation process.

*m*/*z*	Fragment Type	CS-2S	CS-4S	CS-6S
96.4	HSO4^−^	X	X	X
137.0	B^2,5^X_1_	X		
139.0	^1,3^A_1_	X		
157.0	B_1_ (S)/B_1_	X	X	X
175.0	C_1_		X	X
209.0	^1,5^A_1_	X		
237.0	B_1_	X		
255.0	C_1_	X	X	X
354.1	Y_1_	X		
416.1	Z_1_		X	X
434.0	Y_1_		X	X
452.1	^1,3^X_1_	X	X	X
476.1	^0,2^X_1_	X	X	X
492.0	^2,5^X_1_	X		
512.1	M (S)	X	X	X

## Data Availability

Data are contained within the article.
